# Supplementary material for: The DNA methylation landscape of giant viruses
Source: Nat Commun. 2020 May 27;11:2657. doi: 10.1038/s41467-020-16414-2 (PMC7253447; doi:10.1038/s41467-020-16414-2)
Supplement: Supplementary file 1 — Supplementary Information [file 41467_2020_16414_MOESM1_ESM.pdf]

Supplementary Information from Jeudy et al., 2020

The DNA Methylation Landscape of Giant Viruses

Sandra Jeudy[1], Sofia Rigou[1], Jean-Marie Alempic[1], Jean-Michel Claverie[1], Chantal Abergel[1], Matthieu Legendre[1,*]

[1] Aix Marseille Univ., CNRS, IGS, Information Génomique & Structurale (UMR7256), Institut de Microbiologie de la Méditerranée (FR 3489), Marseille, France

* Correspondence to: legendre@igs.cnrs-mrs.fr

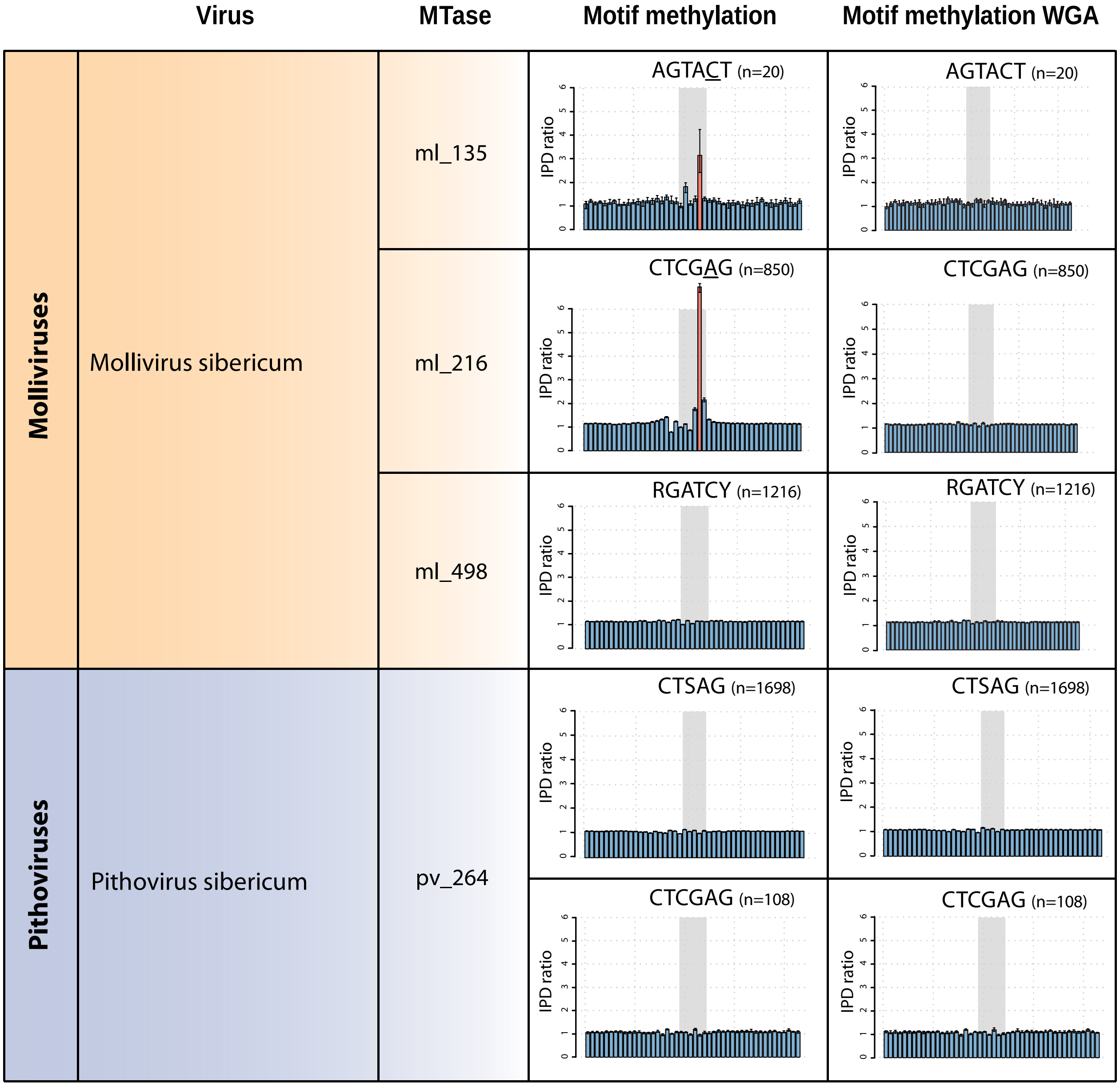

Supplementary Figure 1. IPDr values of mollivirus sibericum and pithovirus sibericum MTases targets from native and WGA DNA

The encoded DNA MTases of both viruses are shown along with their predicted target motifs. Modified nucleotides within the motifs are underlined. Bar graphs correspond to the median IPDr profiles of the corresponding motifs (gray region) and the surrounding 20 nucleotides on each side. Each bar displays the median IPDr value and a 95% confidence interval (error bars) based on 1000 bootstraps. The number of motif occurrences (n) from which these statistics are derived are shown in parenthesis. Red bars correspond to positions with significantly high IPDr values. The IPDr values of native and WGA amplified DNA are shown side by side.

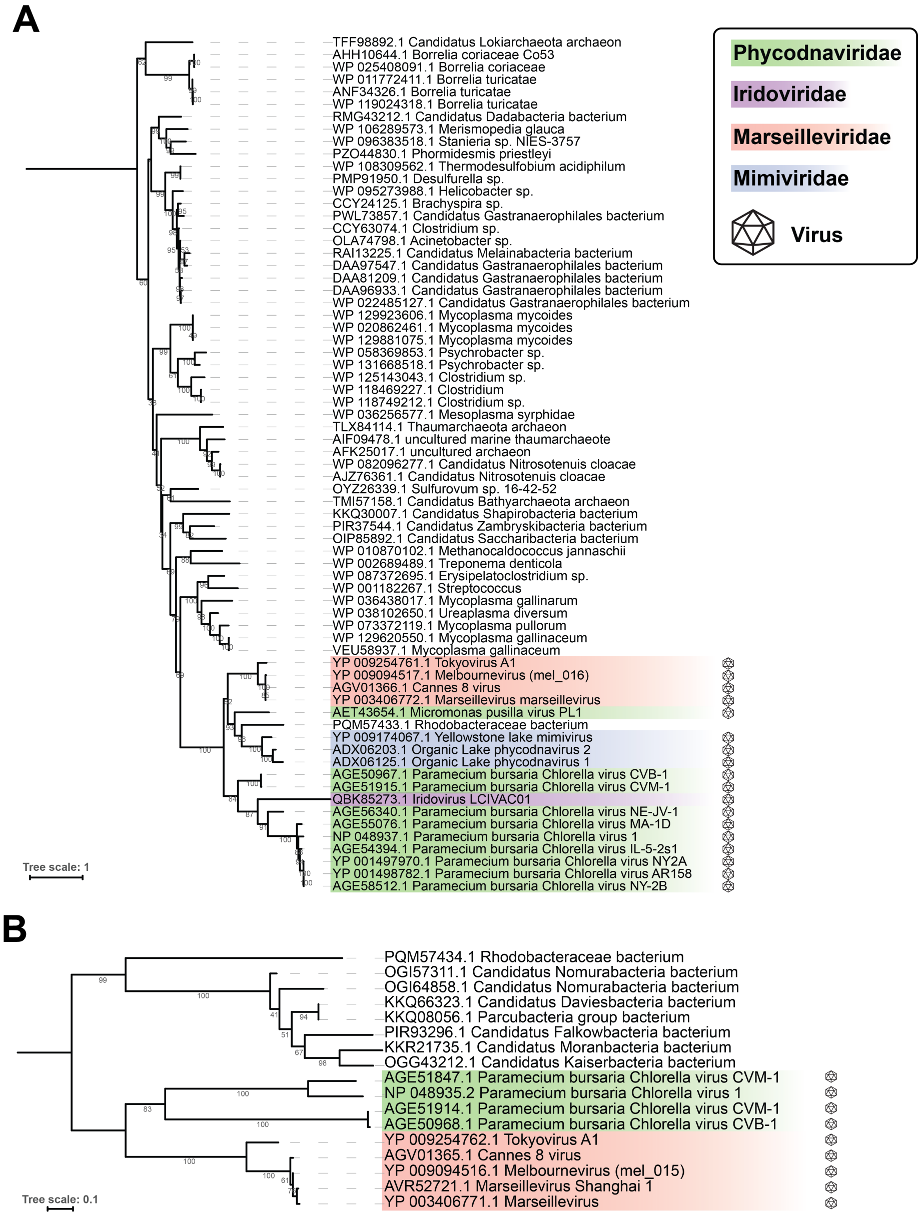

Supplementary Figure 2. Phylogenies of the marseilleviruses R-M system MTase and REase

A) Phylogenetic tree of the mel_016 melbournevirus MTase along with viral and prokaryotic homologs. The tree was calculated using the LG+F+I+G4 model based on a protein sequence alignment of 321 informative sites. Bootstrap values were computed using the UFBoot [1] method from IQtree [2]. The GenBank accessions and taxonomic assignations extracted from GenBank entries are shown. Virion symbols highlight viral sequences that are classified in their corresponding family using a color code described in the inset. Prokaryotic sequences are not color coded. It is worth noting that the Iridovirus sequence comes from a metagenomics assembly [3] and that its taxonomic assignment is uncertain when considering the best blast matchs againt NR of the ORFs encoded in the corresponding genomic contig (Supplementary Figure 8A). Likewise, the *Rhodobacteraceae* bacterial PQM57433.1 sequence also recovered from metagenome assemblies [4] is probably of viral origin (Supplementary Figure 8B). **B**) Similar phylogenetic tree of the mel_015 Melbournevirus MTase. The tree was calculated using the VT+G4 model based on a protein sequence alignment of 188 informative positions.

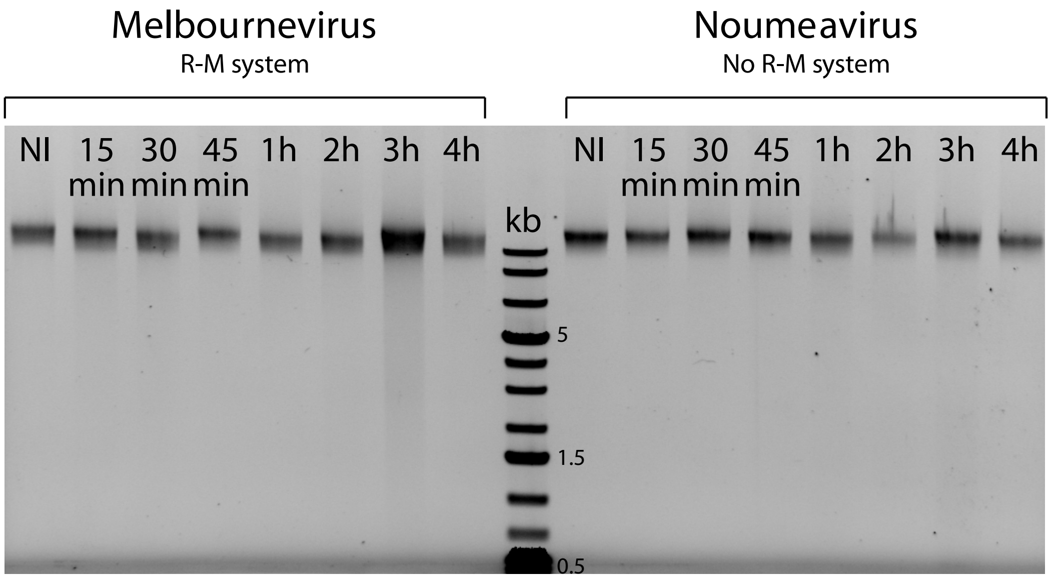

Supplementary Figure 3. Host DNA is not degraded during melbournevirus and noumeavirus infections.

Times (post infection) are listed on the top of the figure. NI corresponds to non-infected. All samples are deposited on the same gel with a molecular weight ladder at lane 9. This experiment was repeated twice with similar results.

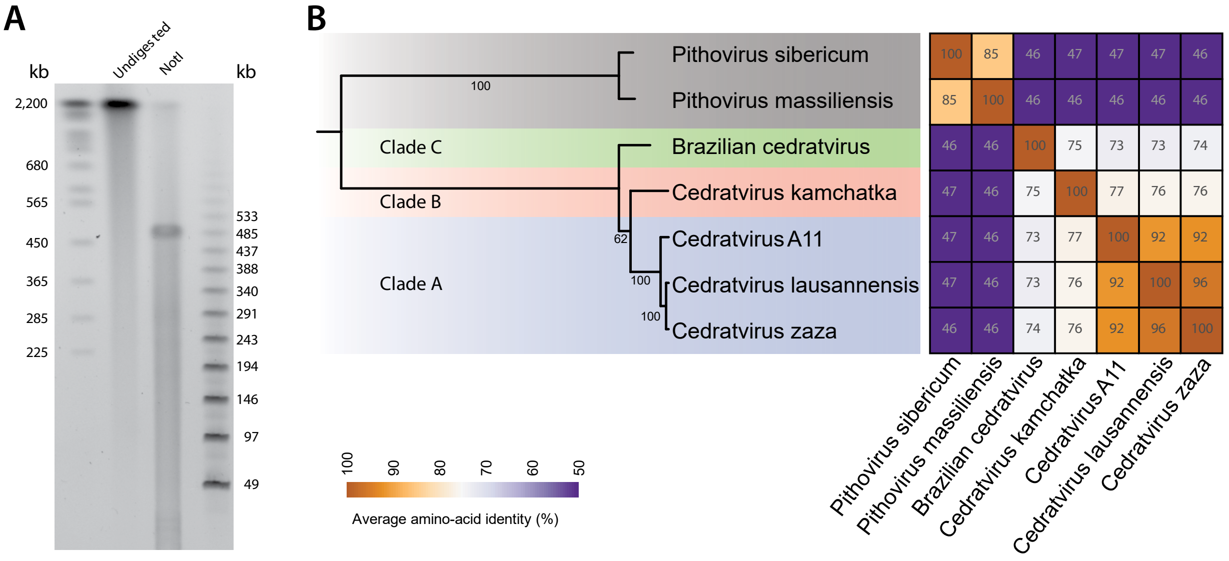

Supplementary Figure 4. Characterization of the cedratviruse kamchatka genome

A) Pulse Field Gel Electrophoresis of the cedratvirus kamchatka genome. The first and last lanes correspond to DNA ladders. Undigested and NotI digested DNA are shown. As expected from its circular structure the undigested DNA migrates higher than the assembled genomic sequence length. The NotI digested DNA, predicted to be cleaved at a single position migrates at the expected position. This experiment was repeated twice with similar results. **B**) Phylogenetic tree of the completely sequenced genomes of cedratviruses and pithoviruses based on the protein sequences alignments of 134 strictly conserved single copy orthologues. The tree was calculated using the best model of each partitioned alignment as determined by IQtree [2]. Bootstrap values (n = 5,000) were computed using the UFBoot [1] method from IQtree [2]. The average amino acid identity matrix was computed using CompareM [5] on the shared ORFs.

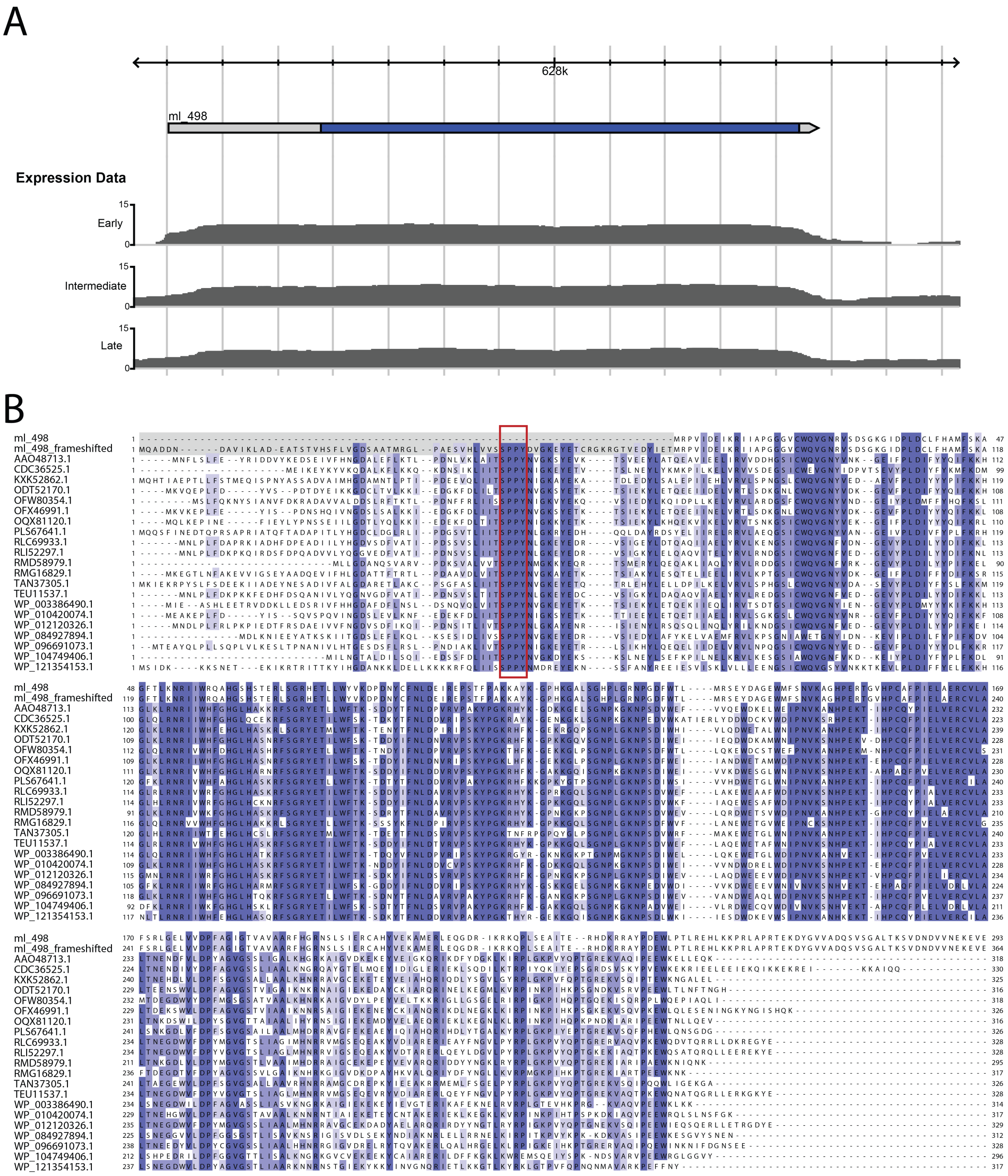

Supplementary Figure 5. Gene structure of the mollivirus sibericum ml_498 gene

A) The structure of the ml_498 gene is shown. Protein coding sequence is in blue and UnTranslated Regions (UTRs) in gray. The transcriptomic RNA-seq data from [6] was mapped to the genome and read density is shown in gray at early, intermediate and late infection time points. **B**) Protein sequence multiple alignment of ml_498 and homologues (named according to their GenBank accession) produced using the Expresso tool [7]. The multiple alignment includes the ml_498 protein sequence as well as a version of the protein sequence were a frameshift in the 5' region of the gene was corrected (ml_498_frameshited). Sequence conservation is highlighted in blue. The red box encompasses a highly conserved xPPY functional motif present in DNA MTases.

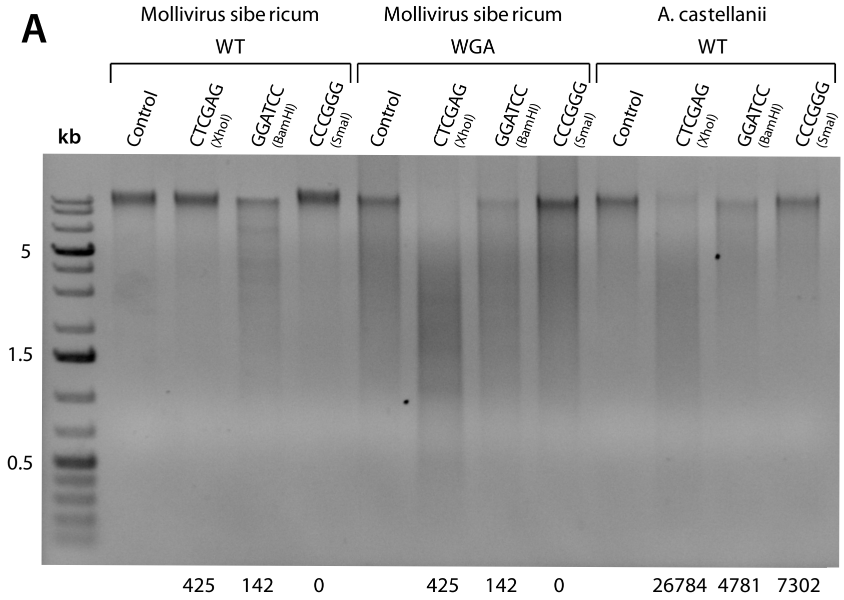

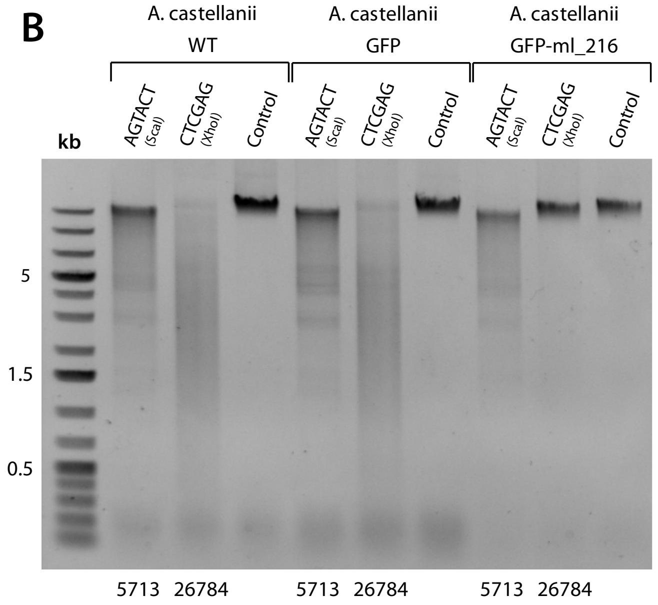

Supplementary Figure 6. Host and mollivirus sibericum DNA methylation assessed by restrictions

A) Agarose gel electrophoresis analysis of mollivirus sibericum and host DNA after digestion with restriction enzymes targeting the same sites as the encoded mollivirus sibericum MTases. Restriction experiments were performed on *A. castellanii* DNA, mollivirus sibericum DNA as well as mollivirus sibericum DNA following whole genome amplification. The theoretical number of fragments expected from the number of occurrences of the motifs in the genomic sequences are noted at the bottom of the figure. **B)** *A. castellanii* host DNA restriction with REases sharing the same targets than ml_216 and ml_135 in wild type conditions and after expression of a GFP protein or a GFP fused with ml_216. These experiments were performed once.

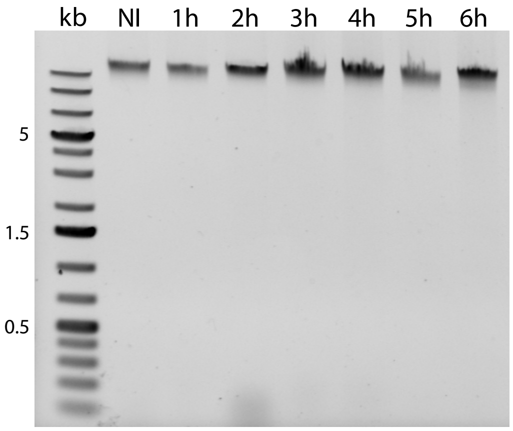

Supplementary Figure 7. Host DNA is not degraded during mollivirus sibericum infection.

Times (post infection) are listed on the top of the figure. NI corresponds to non-infected. This experiment was repeated twice with similar results.

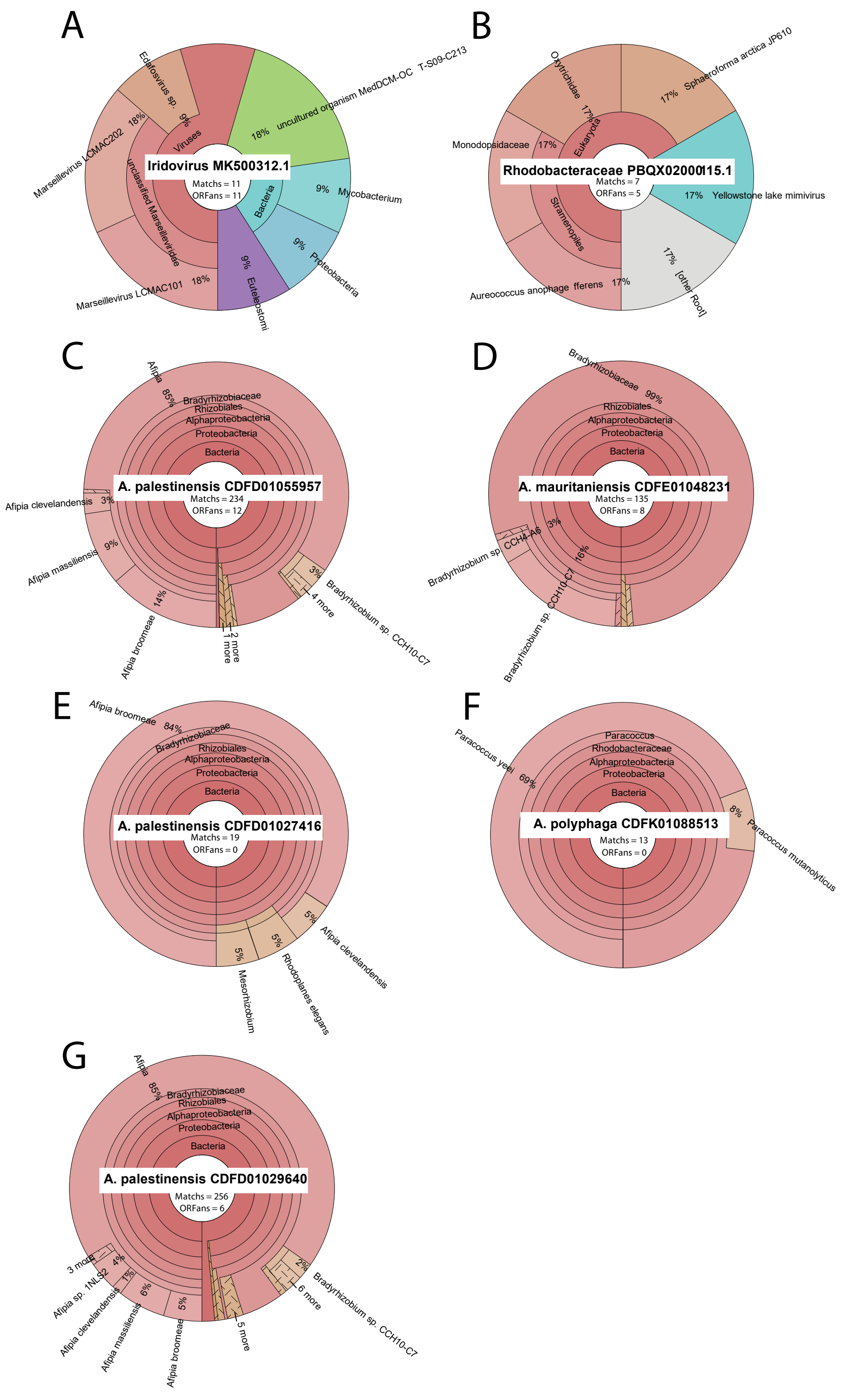

Supplementary Figure 8. Taxonomic assignment of metagenomics and *Acanthamoeba* contigs

For each contig **(A-G)** the taxonomic assignment of the best blastp matchs (with E-value < 10^{-5}) against the NR database of all the encoded ORFs is summarized in a Krona chart [8]. The GenBank accessions of the contigs are displayed as well as the number of ORFs with a significant match and the number of ORFs without match (ORFans).

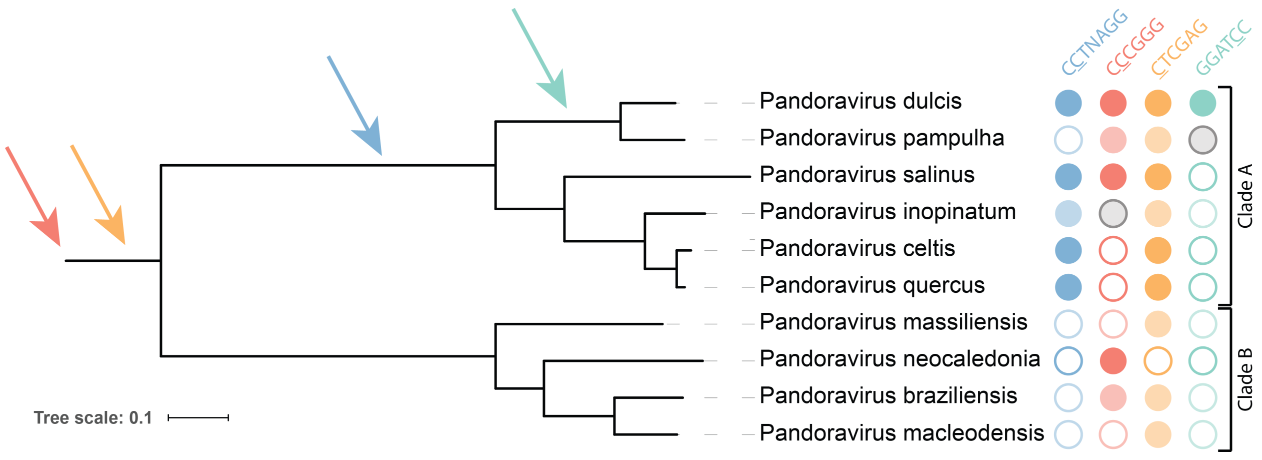

Supplementary Figure 9. Presence/absence of pandoraviruses' MTases mapped onto the pandoraviruses phylogeny

The phylogenetic tree was computed using the protein sequence alignments of 375 strictly conserved single copy orthologues. The tree was calculated using the best model of each partitioned alignment as determined by IQtree [2]. Bootstrap values were computed using the UFBoot [1] method from IQtree [2] but not reported as they were all equal to 100%. For each sub-group of pandoraviruses encoded MTases the presence/absence of the gene is shown with its predicted sequence target using the same colors as in Fig. 5. A filled circle means that a MTase of a given sub group is encoded, while an empty circle means that it is absent. Bold circles highlight MTases for which SMRT data is available while shaded circles depict MTases with no available SMRT data. Gray circles show potential pseudogenes. The colored arrows point to the most parsimonious timings of the MTase acquisition for each sub-group.

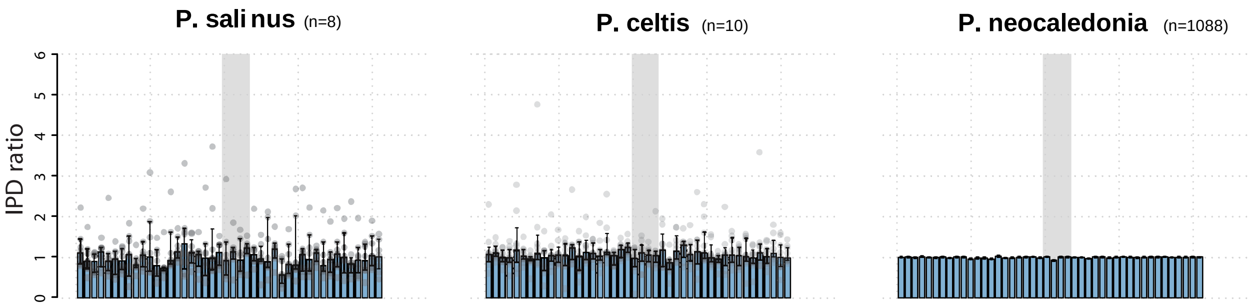

Supplementary Figure 10. Methylation status of the underrepresented AGCT pandoraviruses's tetramer

Median IPDr profiles of the AGCT tetramer (gray region) and the surrounding 20 nucleotides on each side. Each bar displays the median IPDr value and a 95% confidence interval (error bars) based on 1000 bootstraps. The number of motif occurrences (n) from which these statistics are derived are shown in parenthesis. Individual data points are displayed for viruses with n ≤ 10. Pandoravirus salinus and pandoravirus celtis belong to clade A and pandoravirus neocaledonia to clade B [9].

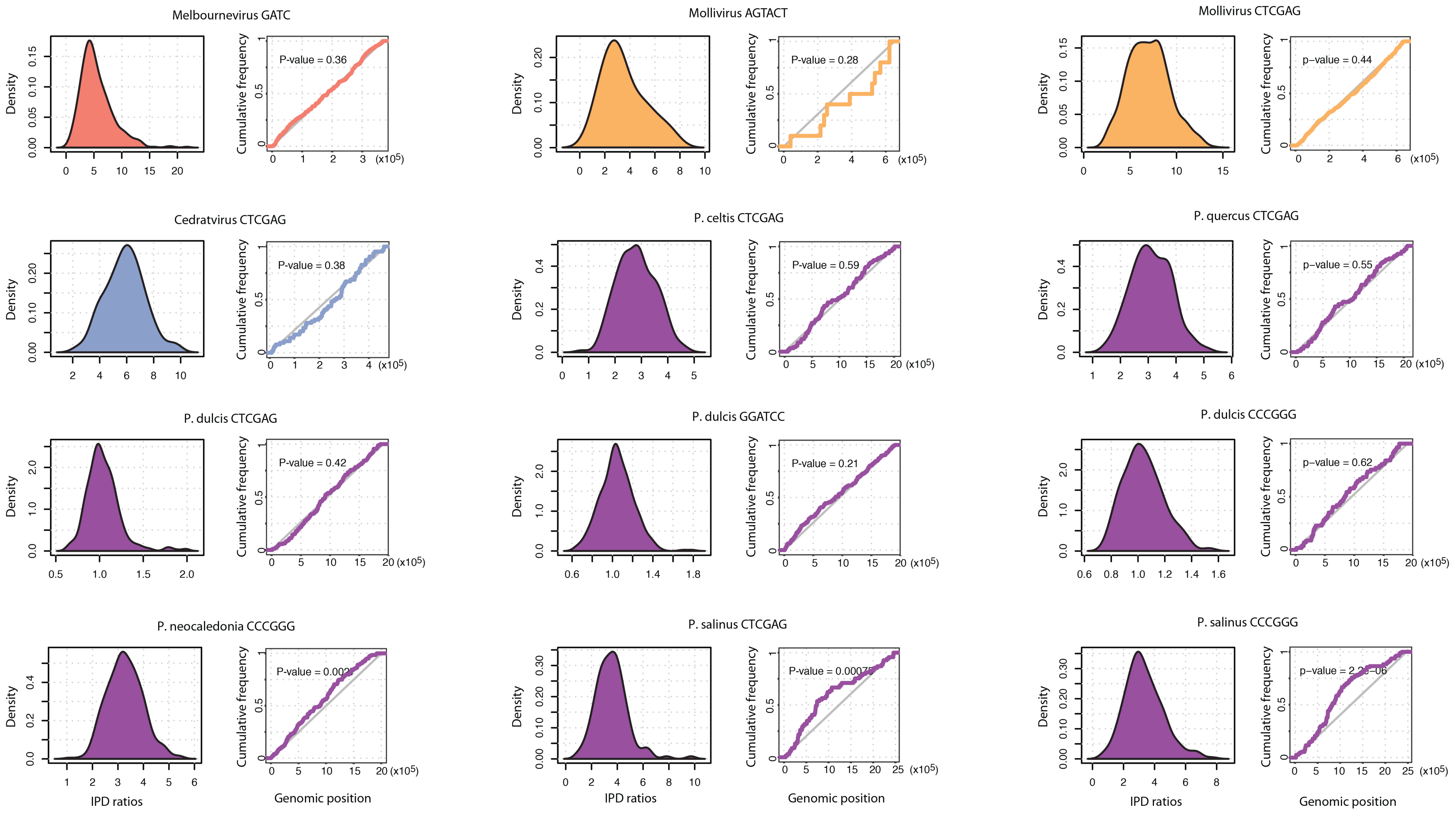

Supplementary Figure 11. Distributions of IPDr values and motifs along the genomes

For each virus and methylated motif pair, we calculated the IPDr values distributions (left graph). We also computed the empirical cumulative distribution of the number of motif occurrence along the genome (right graph). The two-sided Kolmogorov-Smirnov statistical test was used to determine if the motifs were uniformly distributed along the genome. The CCTNAGG motif was excluded from these analyses as there are not sufficient occurrences of this motif in the genomes (see Fig. 1).

Supplementary Table 1. Datasets references

**Virus**	**Reference Genbank ID**	**Reference to the initial SMRT data**	**SMRT data SRR accession**
Megavirus vitis	MG807319	**This study**	SRR11308351
Moumouvirus australiensis	MG807320	**Jeudy et al**. doi: 10.1038/s41396-019-0565-y	SRR11308350
Zamilon zitis	MG807318	**This study**	SRR11308351
Megavirus vitis transpoviron	MG807316	**This study**	SRR11308351
Moumouvirus australiensis transpoviron	MG807317	**Jeudy et al**. doi: 10.1038/s41396-019-0565-y	SRR11308350
Melbournevirus	KM275475	**Philippe N, Legendre M et al.** doi: 10.1126/science.1239181	SRR11308348
Pandoravirus dulcis	KC977570		SRR11308348
Pandoravirus salinus	KC977571		SRR11308347
Pandoravirus quercus	MG011689	**Legendre M et al.** doi: 10.1038/s41467-018-04698-4	SRR11308346
Pandoravirus neocaledonia	MG011690		SRR11310657<br>SRR11308345
Pandoravirus celtis	MK174290	**Legendre M et al.** doi: 10.3389/fmicb.2019.00430	SRR11308344
Mollivirus sibericum	KR921745	**This study**	SRR11308343
Mollivirus sibericum WGA	KR921745	**This study**	SRR11308342
Pithovirus sibericum	KF740664	**This study**	SRR11308341
Pithovirus sibericum WGA	KF740664	**This study**	SRR11308349
Cedratvirus kamtchatka	MN873693	**This study**	SRR11336162

Supplementary Table 2. Protein-coding genes unique to cedratvirus kamchatka (2 pages)

**Gene**	**Predicted function**	**Putative evolutionary scenario**
ck112	Hypothetical protein	*de novo* creation (or HGT from an unknown organism) and loss in Clade A
ck113	Hypothetical protein	Uncertain
ck114	Hypothetical protein	*de novo* creation or HGT from an unknown organism
ck12	Hypothetical protein	Putative HGT
ck137	Hypothetical protein	*de novo* creation or HGT from an unknown organism
ck14	Hypothetical protein	*de novo* creation (or HGT from an unknown organism) and loss in Clade A and C
ck153	Hypothetical membrane protein	*de novo* creation or HGT from an unknown organism
ck154	Hypothetical protein	*de novo* creation (or HGT from an unknown organism) and loss in Clade A and C
ck155	Hypothetical protein	Putative HGT
ck16	Hypothetical protein	*de novo* creation or HGT from an unknown organism
ck17	Hypothetical protein	*de novo* creation or HGT from an unknown organism
ck170	Hypothetical protein	*de novo* creation (or HGT from an unknown organism) and loss in Clade A
ck18	Hypothetical membrane protein	*de novo* creation or HGT from an unknown organism
ck207	Hypothetical protein	*de novo* creation (or HGT from an unknown organism) and loss in Clade A
ck208	Hypothetical membrane protein	*de novo* creation (or HGT from an unknown organism) and loss in Clade A
ck209	Hypothetical membrane protein	*de novo* creation (or HGT from an unknown organism) and loss in Clade A
ck210	Hypothetical membrane protein	*de novo* creation or HGT from an unknown organism
ck211	Hypothetical protein	*de novo* creation (or HGT from an unknown organism) and loss in Clade A
ck213	Hypothetical protein	*de novo* creation or HGT from an unknown organism
ck226	Hypothetical protein	*de novo* creation or HGT from an unknown organism
ck236	Hypothetical membrane protein	Putative HGT
ck237	Hypothetical membrane protein	*de novo* creation or HGT from an unknown organism
ck238	Hypothetical protein	*de novo* creation (or HGT from an unknown organism) and loss in Clade A
ck266	Hypothetical protein	*de novo* creation or HGT from an unknown organism
ck278	Hypothetical protein	*de novo* creation or HGT from an unknown organism
ck312	Hypothetical protein	*de novo* creation (or HGT from an unknown organism) and loss in Clade A
ck345	Hypothetical protein	Putative HGT
ck360	Hypothetical protein	*de novo* creation (or HGT from an unknown organism) and loss in Clade A
ck412	DNA-Adenine methyltransferase	HGT
ck421	Hypothetical protein golgin subfamily A member 6-like	Putative HGT and loss in Clade A
ck423	Hypothetical protein	*de novo* creation (or HGT from an unknown organism) and loss in Clade A and C

ck469	Hypothetical protein	*de novo* creation or HGT from an unknown organism
Ck471	Hypothetical membrane protein	*de novo* creation (or HGT from an unknown organism) and loss in Clade A and C
ck472	Hypothetical protein	*de novo* creation (or HGT from an unknown organism) and loss in Clade A
ck473	Hypothetical membrane protein	*de novo* creation (or HGT from an unknown organism) and loss in Clade A
ck474	Hypothetical membrane protein	*de novo* creation (or HGT from an unknown organism) and loss in Clade A
ck476	Hypothetical protein	*de novo* creation (or HGT from an unknown organism) and loss in Clade A
ck477	Hypothetical membrane protein	*de novo* creation (or HGT from an unknown organism) and loss in Clade A
ck478	Hypothetical membrane protein	*de novo* creation (or HGT from an unknown organism) and loss in Clade A and C
ck479	Hypothetical protein	*de novo* creation or HGT from an unknown organism *de novo* creation (or HGT from an unknown organism) and loss in Clade A
ck480	Hypotetical membrane protein	*de novo* creation (or HGT from an unknown organism) and loss in Clade A
ck481	Hypothetical protein	*de novo* creation (or HGT from an unknown organism) and loss in Clade A
ck52	Hypothetical protein	*de novo* creation or HGT from an unknown organism
ck522	Hypothetical protein	*de novo* creation or HGT from an unknown organism
ck523	Hypothetical protein	*de novo* creation or HGT from an unknown organism
ck524	Hypothetical protein	*de novo* creation (or HGT from an unknown organism) and loss in Clade A
ck525	Hypothetical membrane protein	*de novo* creation or HGT from an unknown organism
ck526	Hypothetical protein	*de novo* creation or HGT from an unknown organism
ck90	Hypothetical protein	*de novo* creation or HGT from an unknown organism
ck95	Hypothetical protein	*de novo* creation or HGT from an unknown organism
ck96	Hypothetical membrane protein	Putative HGT

Supplementary Table 3. RNA-seq transcriptomic data from [10] of the pithovirus sibericum pv_264 MTase

	**pv_264 mapped reads**	**FPKM**	**Gene expression rank (over 467 P. sibericum genes)**
Non infected	0	0	NA
4h pi	6	62.7	181
11h pi	5	82.9	221
16h pi	8	454.2	107

Supplementary Table 4. RNA-seq transcriptomic data from [10] of the pithovirus sibericum pv_113 MTase

	**pv_113 mapped reads**	**FPKM**	**Gene expression rank (over 467 P. sibericum genes)**
Non infected	6	0	NA
4h pi	65	78.2	41
11h pi	38	72.4	51
16h pi	15	103.2	67

Supplementary Table 5. RNA-seq transcriptomic data from [6] of the mollivirus sibericum ml_498 MTase

	**ml_498 mapped reads**	**FPKM**	**Gene expression rank (over 523 M. sibericum genes)**
30min-1h-2h pi	8982	168.7	149
3h-4h-5h pi	15593	252.2	383
6h-7h-9h pi	7187	113.6	398

Supplementary Table 6. dN/dS ratios of MTases

Virus	Gene	dN/dS*	Model	B_free vs B_neut model P-value#	Viral DNA Methylation
Mollivirus sibericum	ml_135	-	-	-	Yes
	ml_216	-	-	-	Yes
	ml_498	-	-	-	No, pseudogene
Cedratvirus kamtchatka	ck412	-	-	-	Yes
Pithovirus sibericum	pv_264	-	-	-	No
Pandoravirus salinus	psal_cds_866	<u>0.281</u>	b_free	0.00006	Yes
	psal_cds_930	1	b_neut	0.1	Yes
	psal_cds_656	0.354	b_neut	0.19	Yes
Pandoravirus dulcis	pdul_cds_639	1	b_neut	0.21	Yes
	pdul_cds_713	<u>0.204</u>	b_free	0.014	Yes
	pdul_cds_889	0.320	b_neut	0.31	Yes
	pdul_cds_425	0.658	b_neut	0.42	Yes
Pandoravirus inopinatum	pino_cds_419	-	-	-	Pseudogene
	pino_cds_741	<u>0.28</u>	b_free	0.0001	-
	pino_cds_313	0.624	b_neut	0.66	-
Pandoravirus pampulha	ppam_cds_637	0.493	b_neut	0.44	-
	ppam_cds_578	-	-	-	Pseudogene
	ppam_cds_790	0.529	b_neut	0.8	-
Pandoravirus quercus	pqer_cds_896	<u>0.242</u>	b_free	0.007	Yes
	pqer_cds_559	<u>0.144</u>	M0	-	Yes
Pandoravirus celtis	pcts_cds_910	<u>0.361</u>	b_free	0.02	Yes
	pcts_cds_576	<u>0.144</u>	M0	-	Yes
Pandoravirus neocaledonia	pneo_cds_78	<u>0.272</u>	b_free	0.04	Yes
Pandoravirus braziliansis	pbra_cds_857	0.357	b_neut	0.21	-
	pbra_cds_273	<u>0.243</u>	b_free	0.0003	-
Pandoravirus massiliensis	pmas_cds_277	<u>0.164</u>	b_free	0.0002	-
Pandoravirus macleodensis	pmac_cds_678	<u>0.304</u>	b_free	0.014	-

* dN/dS significantly different from 1 are underlined. dN/dS of pseudogenes and genes with saturation in substitutions (dS > 1.5) were not computed.

\# P-values were computed using likelihood-ratio chi-squared tests. No multiple testing adjustment was performed.

Supplementary References

1. Minh, B. Q., Nguyen, M. A. T. & von Haeseler, A. Ultrafast Approximation for Phylogenetic Bootstrap. *Mol Biol Evol* **30**, 1188–1195 (2013).

2. Nguyen, L.-T., Schmidt, H. A., von Haeseler, A. & Minh, B. Q. IQ-TREE: a fast and effective stochastic algorithm for estimating maximum-likelihood phylogenies. *Mol. Biol. Evol.* **32**, 268–274 (2015).

3. Bäckström, D. *et al.* Virus Genomes from Deep Sea Sediments Expand the Ocean Megavirome and Support Independent Origins of Viral Gigantism. *MBio* **10**, (2019).

4. Graham, E. D., Heidelberg, J. F. & Tully, B. J. Potential for primary productivity in a globally-distributed bacterial phototroph. *ISME J* **12**, 1861–1866 (2018).

5. Parks, D. *A toolbox for comparative genomics. Contribute to dparks1134/CompareM development by creating an account on GitHub*. (2019).

6. Legendre, M. *et al.* In-depth study of Mollivirus sibericum, a new 30,000-y-old giant virus infecting Acanthamoeba. *Proc. Natl. Acad. Sci. U.S.A.* **112**, E5327-5335 (2015).

7. Armougom, F. *et al.* Expresso: automatic incorporation of structural information in multiple sequence alignments using 3D-Coffee. *Nucleic Acids Res.* **34**, W604-608 (2006).

8. Ondov, B. D., Bergman, N. H. & Phillippy, A. M. Interactive metagenomic visualization in a Web browser. *BMC Bioinformatics* **12**, 385 (2011).

9. Legendre, M. *et al.* Pandoravirus celtis illustrates the microevolution processes at work in the giant Pandoraviridae genomes. *Front. Microbiol.* **10**, (2019).

10. Legendre, M. *et al.* Thirty-thousand-year-old distant relative of giant icosahedral DNA viruses with a pandoravirus morphology. *Proc. Natl. Acad. Sci. U.S.A.* **111**, 4274–4279 (2014).
