# Supplementary material for: The DNA methylation landscape of giant viruses
Source: Nat Commun. 2020 May 27;11:2657. doi: 10.1038/s41467-020-16414-2 (PMC7253447; doi:10.1038/s41467-020-16414-2)
Supplement: Supplementary file 3 — Reporting Summary [file 41467_2020_16414_MOESM3_ESM.pdf]

nature research

Corresponding author(s): Matthieu Legendre
Last updated by author(s): 2020-05-01

Reporting Summary

Nature Research wishes to improve the reproducibility of the work that we publish. This form provides structure for consistency and transparency in reporting. For further information on Nature Research policies, see Authors & Referees and the Editorial Policy Checklist .

Statistics

For all statistical analyses, confirm that the following items are present in the figure legend, table legend, main text, or Methods section.

n/a	Confirmed	
	x	The exact sample size (*n*) for each experimental group/condition, given as a discrete number and unit of measurement
x		A statement on whether measurements were taken from distinct samples or whether the same sample was measured repeatedly
	x	The statistical test(s) used AND whether they are one- or two-sided<br>*Only common tests should be described solely by name; describe more complex techniques in the Methods section.*
x		A description of all covariates tested
x		A description of any assumptions or corrections, such as tests of normality and adjustment for multiple comparisons
	x	A full description of the statistical parameters including central tendency (e.g. means) or other basic estimates (e.g. regression coefficient) AND variation (e.g. standard deviation) or associated estimates of uncertainty (e.g. confidence intervals)
	x	For null hypothesis testing, the test statistic (e.g. *F*, *t*, *r*) with confidence intervals, effect sizes, degrees of freedom and *P* value noted<br>*Give P values as exact values whenever suitable.*
x		For Bayesian analysis, information on the choice of priors and Markov chain Monte Carlo settings
x		For hierarchical and complex designs, identification of the appropriate level for tests and full reporting of outcomes
x		Estimates of effect sizes (e.g. Cohen's *d*, Pearson's *r*), indicating how they were calculated

Our web collection on statistics for biologists contains articles on many of the points above.

Software and code

Policy information about availability of computer code

Data collection	No software was used for data collection.
Data analysis	Cedratvirus kamchatka genome was assembled using Flye version 2.4.2 and annotated using Genemark-S version 4.30. SMRT-seq data was analyzed using the SMRTanalysis package version 2.3.0. Protein multiple alignments were performed using: T-coffee version 12.00.7fb08c2 with the Mcoffee and Expresso options, Mafft version 7.407, Clustal Omega version 1.2.4 and analyzed using TrimAl version 1.2rev59. Homology searches were performed using the online version of Blast as well as the command line version 2.8.1. Phylogenies and comparative genomics were done using IQtree version 1.6.2, OrthoFinder version 2.3.10, ETE3 version 3.1.1 and CompareM version 0.0.23.

For manuscripts utilizing custom algorithms or software that are central to the research but not yet described in published literature, software must be made available to editors/reviewers. We strongly encourage code deposition in a community repository (e.g. GitHub). See the Nature Research guidelines for submitting code & software for further information.

Data

Policy information about availability of data

All manuscripts must include a data availability statement. This statement should provide the following information, where applicable:

- Accession codes, unique identifiers, or web links for publicly available datasets
- A list of figures that have associated raw data
- A description of any restrictions on data availability

Data supporting the findings of this work are available within the paper and its Supplementary Information files. The raw SMRT sequence datasets generated and analyzed in the current study were deposited in the Sequence Read Archive database under the following accession PRJNA612691 [https://www.ncbi.nlm.nih.gov/bioproject/612691]. In addition individual datasets accessions are all reported in the Supplementary Table 1. The assembled cedratvirus kamchatka genome has been deposited to the GenBank database under the following accession MN873693 [https://www.ncbi.nlm.nih.gov/nuccore/MN873693]. MTases annotations were performed using the REbase database [http://rebase.neb.com/rebase/rebase.html] and the cedratvirus kamchatka gene annotations using the Blast NCBI NR (GenBank CDS translations+PDB+SwissProt +PIR+PRF) database and the Uniprot-Swissprot [https://www.uniprot.org/uniprot/] database. The source data underlying Figure 1, Supplementary Figure 1 and 10, Supplementary Tables 3, 4 and 5 are provided as Source Data file.

Field-specific reporting

Please select the one below that is the best fit for your research. If you are not sure, read the appropriate sections before making your selection.

- [x] Life sciences
- [ ] Behavioural & social sciences
- [ ] Ecological, evolutionary & environmental sciences

For a reference copy of the document with all sections, see nature.com/documents/nr-reporting-summary-flat.pdf

Life sciences study design

All studies must disclose on these points even when the disclosure is negative.

Sample size	No sample size calculation was performed but since each genome was sequenced only once but the read coverage was sufficient to statistically identify modified nucleotides.
Data exclusions	No data were excluded.
Replication	All experiments involving agarose gels were performed at least twice with similar results, expect for one that was performed only once and mentioned in the legend. SMRT sequencing was performed only once for each genome but control experiments with WGA DNA were also done for two viruses.
Randomization	For motif enrichment analysis of intergenic regions a shuffling of protein-coding genes coordinates was performed 1000 times to compute empirical p-values.
Blinding	We did not employ blinding in this study since we did not have treatment groups.

Reporting for specific materials, systems and methods

We require information from authors about some types of materials, experimental systems and methods used in many studies. Here, indicate whether each material, system or method listed is relevant to your study. If you are not sure if a list item applies to your research, read the appropriate section before selecting a response.

Materials & experimental systems

n/a	Involved in the study	
x		Antibodies
	x	Eukaryotic cell lines
x		Palaeontology
x		Animals and other organisms
x		Human research participants
x		Clinical data

Methods

n/a	Involved in the study	
x		ChIP-seq
x		Flow cytometry
x		MRI-based neuroimaging

Eukaryotic cell lines

Policy information about cell lines

Cell line source(s)	ATCC 30010
Authentication	None of the cell lines used were authenticated
Mycoplasma contamination	The cell lines were not tested for mycoplasma contamination
Commonly misidentified lines (See ICLAC register)	*Name any commonly misidentified cell lines used in the study and provide a rationale for their use.*
