# Supplementary material for: The DNA methylation landscape of giant viruses
Source: Nat Commun. 2020 May 27;11:2657. doi: 10.1038/s41467-020-16414-2 (PMC7253447; doi:10.1038/s41467-020-16414-2)
Supplement: Supplementary file 4 — Description of Additional Supplementary Files [file 41467_2020_16414_MOESM4_ESM.pdf]

Description of Additional Supplementary Files

File Name: Supplementary Data 1
Description: Clusters of single copy orthologues used to compute pandoraviruses and marseilleviruses phylogenies.
